# Supplementary material for: Waveform detection by deep learning reveals multi-area spindles that are selectively modulated by memory load
Source: eLife. 2022 Jun 29;11:e75769. doi: 10.7554/eLife.75769 (PMC9242645; doi:10.7554/eLife.75769)
Supplement: Transparent reporting form [file elife-75769-transrepform1.pdf]

Westbrook Centre
Milton Road
Cambridge CB4 1YG, UK

P 01223 855340
W elifesciences.org
T @elife

eLife's transparent reporting form

We encourage authors to provide detailed information *within their submission* to facilitate the interpretation and replication of experiments. Authors can upload supporting documentation to indicate the use of appropriate reporting guidelines for health-related research (see EQUATOR Network), life science research (see the BioSharing Information Resource), or the ARRIVE guidelines for reporting work involving animal research. Where applicable, authors should refer to any relevant reporting standards documents in this form.

If you have any questions, please consult our Journal Policies and/or contact us: editorial@elifesciences.org.

Sample-size estimation

- You should state whether an appropriate sample size was computed when the study was being designed
- You should state the statistical method of sample size computation and any required assumptions
- If no explicit power analysis was used, you should describe how you decided what sample (replicate) size (number) to use

Please outline where this information can be found within the submission (e.g., sections or figure legends), or explain why this information doesn't apply to your submission:

We do not make an explicit justification of the sample size for these recordings; rather, in this research, we strove to utilize every recording presently available. The analysis is performed across three sleep datasets. The first dataset contains more than 9 hours of ECoG recordings in two macaques obtained during 7 recording sessions. The second dataset contains EEG recordings from 20 healthy subjects. Each subject participated in two sessions with sleep recordings of 30 or 60 min. The last dataset contains iEEG recordings from 5 epileptic patients. Each patient is hospitalized for 7 to 14 days with upto 3 clinically annotated sleeps per day. In total, we analyzed 89 30-min sleep recordings extracted from these annotations. All these recordings provide many hours of sleep for which population statistics are thoroughly reported to justify our results.

eLife Sciences Publications, Ltd is a limited liability non-profit non-stock corporation incorporated in the State of Delaware, USA, with company number 5030732, and is registered in the UK with company number FC030576 and branch number BR015634 at the address Westbrook Centre, Milton Road, Cambridge CB4 1YG.

Replicates

- You should report how often each experiment was performed
- You should include a definition of biological versus technical replication
- The data obtained should be provided and sufficient information should be provided to indicate the number of independent biological and/or technical replicates
- If you encountered any outliers, you should describe how these were handled
- Criteria for exclusion/inclusion of data should be clearly stated
- High-throughput sequence data should be uploaded before submission, with a private link for reviewers provided (these are available from both GEO and ArrayExpress)

Please outline where this information can be found within the submission (e.g., sections or figure legends), or explain why this information doesn't apply to your submission:

We analyzed all sleep recordings across all sleep datasets except in EEG dataset in which we only include 16 out of 20 participants. The four subjects excluded from analysis had sleep recordings that did not reach stage 2 sleep or were too noisy (lines 252-253).

eLife Sciences Publications, Ltd is a limited liability non-profit non-stock corporation incorporated in the State of Delaware, USA, with company number 5030732, and is registered in the UK with company number FC030576 and branch number BR015634 at the address Westbrook Centre, Milton Road, Cambridge CB4 1YG.

Statistical reporting

- Statistical analysis methods should be described and justified
- Raw data should be presented in figures whenever informative to do so (typically when N per group is less than 10)
- For each experiment, you should identify the statistical tests used, exact values of N, definitions of center, methods of multiple test correction, and dispersion and precision measures (e.g., mean, median, SD, SEM, confidence intervals; and, for the major substantive results, a measure of effect size (e.g., Pearson's r, Cohen's d)
- Report exact p-values wherever possible alongside the summary statistics and 95% confidence intervals. These should be reported for all key questions and not only when the p-value is less than 0.05.

Please outline where this information can be found within the submission (e.g., sections or figure legends), or explain why this information doesn't apply to your submission:

In this submission statistical tests are reported at seven key points: (1) The comparison of amplitude of spindles detected by AT and CNN approach (one-sided Wilcoxon signed-rank test, lines 117-118), (2) The comparison of the increase in the multi-area spindles detected by the CNN vs local spindles (one-sided Wilcoxon signed-rank test, lines 134-135), (3) The comparison of the increase in the regional spindles detected by the CNN vs local spindles (one-sided Wilcoxon signed-rank test, lines 136-137), (4) The comparison of the amplitudes of detected spindles in low and high visual working memory conditions (Wilcoxon signed-rank test, lines 158-159), (5) The comparison of the increase in the number multi-area spindles detected by CNN model in high- vs low-working memory conditions (one-sided paired-sample t-test, lines 161-162, and Figure 3), (6) The comparison of the increase in the number multi-area spindles detected by SNR model in high- vs low-working memory conditions (one-sided paired-sample t-test, Supplementary Figure 4a), (7) The comparison of the increase in the number multi-area spindles detected by AT model in high- vs low-working memory conditions (one-sided paired-sample t-test, Supplementary Figure 4b). Exact p-values are reported except where they become extremely small.

(For large datasets, or papers with a very large number of statistical tests, you may upload a single table file with tests, Ns, etc., with reference to sections in the manuscript.)

Group allocation

- Indicate how samples were allocated into experimental groups (in the case of clinical studies, please specify allocation to treatment method); if randomization was used, please also state if restricted randomization was applied
- Indicate if masking was used during group allocation, data collection and/or data analysis

Please outline where this information can be found within the submission (e.g., sections or figure legends), or explain why this information doesn't apply to your submission:

We performed our analysis over three sleep datasets obtained from different electrode types. Due to the differences across recording electrodes, we decided to perform our analysis and statistical tests over recordings within each recording type. We achieved similar results across all datasets, and no further group allocation is performed within each dataset through the analysis.

Additional data files ("source data")

- We encourage you to upload relevant additional data files, such as numerical data that are represented as a graph in a figure, or as a summary table

- Where provided, these should be in the most useful format, and they can be uploaded as "Source data" files linked to a main figure or table
- Include model definition files including the full list of parameters used
- Include code used for data analysis (e.g., R, MatLab)
- Avoid stating that data files are "available upon request"

Please indicate the figures or tables for which source data files have been provided:

Source data for non human primate electrocorticographic (ECoG) recording and human EEG recordings were made freely available through following links:
http://neurotycho.org/sleep-task
https://osf.io/chav7

Our custom MATLAB (MathWorks) implementations of all computational analyses, along with the analysis scripts used for this study will be made available as an open-access release on GitHub (http://mullerlab.github.io).

eLife Sciences Publications, Ltd is a limited liability non-profit non-stock corporation incorporated in the State of Delaware, USA, with company number 5030732, and is registered in the UK with company number FC030576 and branch number BR015634 at the address Westbrook Centre, Milton Road, Cambridge CB4 1YG.
